# The Crossroads of Neurodegeneration: Exploring the Overlap Between Alzheimer's Disease and Depression

**DOI:** 10.7759/cureus.104771

**Published:** 2026-03-06

**Authors:** Prayna Bhatia, Justin Peter Rosales, Pooja Patel, Gabrielle Brini, Jake Singh, Raj Patel, Jack Petroski, Benjamin Brisman, Manveer Sandhu, Nicholas Santacroce, Adityabikram Singh, Gurjinder Kaur

**Affiliations:** 1 Neurology, Touro College of Osteopathic Medicine, Middletown, USA; 2 Urology, Lenox Hill Hospital, New York City, USA; 3 Physiology, Touro College of Osteopathic Medicine, Middletown, USA

**Keywords:** alzheimer's disease, depression, mitochondrial dysfunction, oxidative stress, vascular dysfunction

## Abstract

Alzheimer’s disease (AD) and depression are two prevalent and debilitating neuropsychiatric disorders that frequently occur together and share overlapping clinical and molecular features. Understanding shared features of AD and depression is critical for delaying disease progression and improving quality of life. The objective of this narrative review is to identify recent advances in the understanding of AD and its link to depression, with a focus on identifiable biomarkers.

This review explores the converging pathophysiological pathways implicated in both AD and depression, with an emphasis on oxidative stress, mitochondrial dysfunction, microglial activation, vascular impairment, and metal ion dysregulation. Specific attention is given to biomarkers of lipid peroxidation, nucleic acid damage, and protein nitration, as well as to the role of telomerase activity and ferroptosis in neuronal cell death. Additionally, we evaluate the therapeutic potential of antidepressants in modifying disease trajectory and reducing depressive symptoms in AD patients. By investigating the molecular intersections of AD and depression, this review aims to provide a comprehensive understanding of their shared pathology and highlight new avenues for targeted interventions. Our review underscores the overlapping mechanisms between AD and depression, paving the way for earlier detection, targeted therapies, closer monitoring, and improved patient outcomes.

## Introduction and background

Alzheimer’s disease (AD) is more than a memory disorder; rather, it is a progressive neurodegenerative disorder that initially manifests as mild cognitive impairment and quickly advances to neuropsychiatric disturbances such as behavioral and psychological symptoms. Classified as a major public health problem, AD occupies the seventh position among the leading causes of mortality in the United States, affecting an estimated 6.8 million Americans and impacting 36.5 million individuals worldwide [1,2]. Most often, AD presents with clinical features encompassing memory deficits, poor judgment, speech difficulties, compromised visuospatial orientation, and motor dysfunction, which ultimately result in mood and personality changes, confusion, and social withdrawal [3,4]. Since the onset of cognitive decline, approximately 90% of Alzheimer’s patients experience at least one form of neuropsychiatric symptom, including psychosis, agitation, aggression, anxiety, apathy, and depressive mood [5-7]. Studies have shown that the presence of neuropsychiatric symptoms in AD could alter disease severity, treatment response, and overall prognosis, resulting in numerous adverse outcomes for patients and their caregivers [7,8].

The overlap between symptoms in depression and AD makes early, precise detection and treatment inefficient. If left unresolved, it can accelerate disease progression and result in early institutionalization of patients, contributing to a substantial economic and societal burden on the healthcare system. Little is known about the pathophysiology and link between depression and Alzheimer’s. However, due to the debilitating impact of depression on the quality of life of patients with AD, a better understanding of the relationship between AD and depression might be of clinical importance.

**Figure 1:**
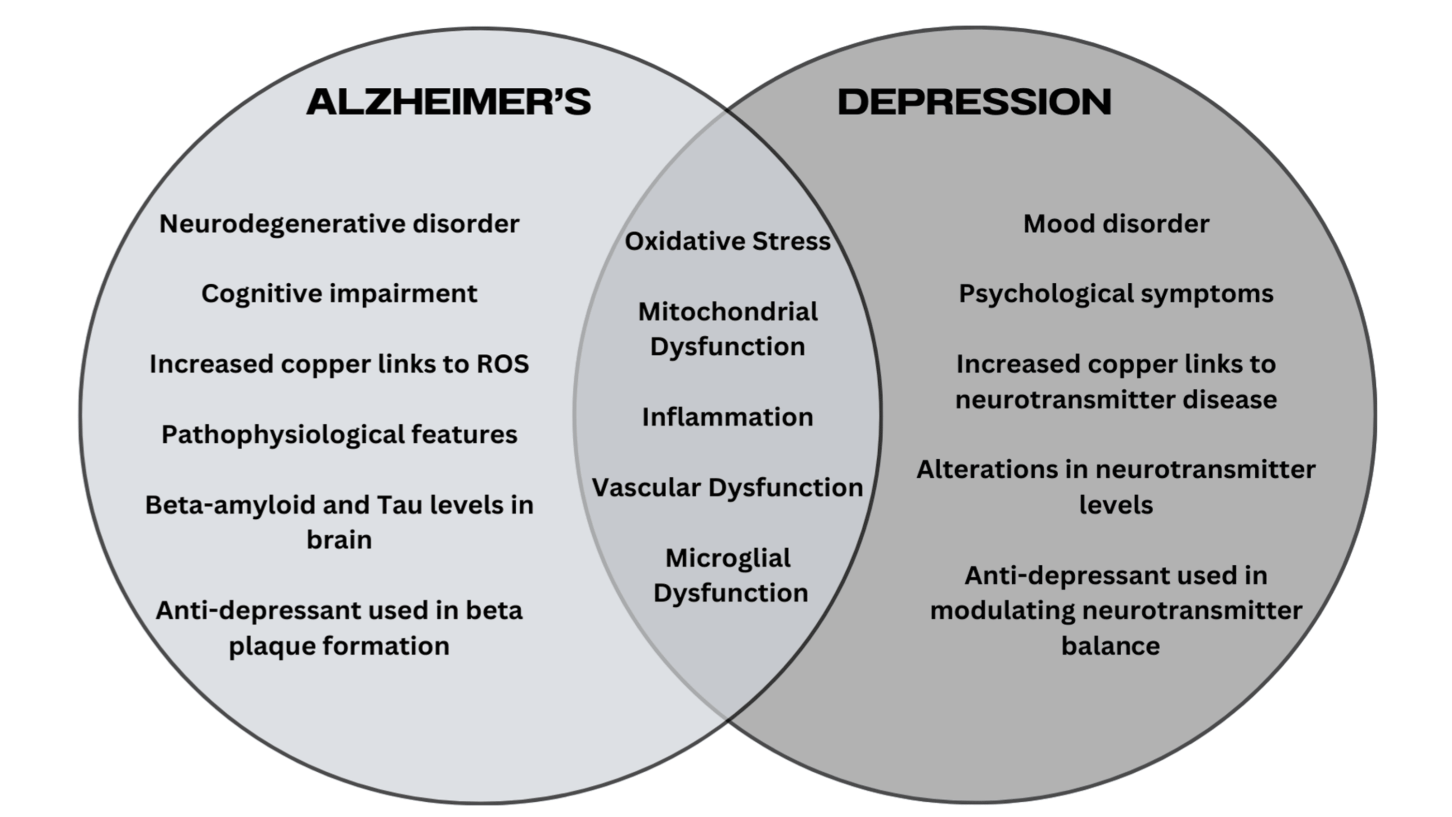
Alzheimer’s disease, depression, and overlapping pathophysiological mechanisms Source: Adapted from [5,6,8,9] This image was created by the authors using Canva (Canva Pty Ltd, Sydney, Australia). ROS, reactive oxygen species

This narrative review aims to unravel the shared pathophysiological mechanisms between AD and depression. This review addresses how oxidative stress (cellular damage), mitochondrial dysfunction (energy failure in cells), inflammation, vascular dysfunction (blood flow issues), and microglial dysfunction (leading to brain inflammation) are linked to both AD and depression. Furthermore, we explore how elements such as copper, neurotransmitters, and antidepressants have shared pathophysiology and effects in AD and depression. Figure 1 outlines the roadmap of the paper and the shared characteristics of both AD and depression. We focus on the converging molecular, cellular, and neuroanatomical alterations that contribute to their co-occurrence and mutual exacerbation, with the goal of identifying potential targets for more effective therapeutic interventions.

## Review

Methods

A comprehensive literature search was conducted using PubMed/MEDLINE and PsycINFO for papers published between 1995 and 2025. Search strategies employed both Medical Subject Headings (MeSH) and text word searches, combining terms related to AD, dementia, depression, and mood disorders using Boolean operators. Inclusion criteria encompassed peer-reviewed original research articles, systematic reviews, and meta-analyses examining the relationship between AD and depression in adult populations.

Oxidative stress

Increased reactive oxygen species (ROS) and reactive nitrogen species, resulting in oxidative stress, are prevalent biological markers encountered in both depression and AD. Oxidative stress is a result of an imbalance between naturally occurring oxidants and antioxidants in which free radicals overwhelm antioxidant protection [5]. When present at normal levels, ROS play a critical physiological role as signaling molecules in various redox-dependent signaling pathways of cell growth, differentiation, and death, but at elevated levels, they can become quite damaging to the human body, inducing molecular pathogenesis [6]. The principal mechanisms responsible for increasing ROS in neurological disorders are overactivation of oxidases [7,8], mitochondrial dysfunction [10], and disruption of metal ion homeostasis [11]. Several studies show evidence of oxidative stress in postmortem AD and depression brain tissue, indicative of oxidation of biomolecules, generating lipid peroxidation, nucleic acid damage, and protein modification [12].

Lipid peroxidation

Excess free radicals attack the cell membranes and lead to lipid peroxidation, which is a chain reaction involving the oxidation of polyunsaturated fatty acids such as arachidonic acid. Propagation of lipid peroxidation can lead to cell death via apoptosis, autophagy, and ferroptosis [9]. Ferroptosis is a type of cell death pathway in which iron dysregulation leads to a decrease in the activity level of glutathione peroxidase, an enzyme essential for the detoxification of hydroperoxides in membrane lipids [13]. Neuronal cell death by ferroptosis causes the release of immunogenic lipid metabolites, initiating inflammation in the brain, which is a pathological feature of both depression and AD [13].

F2-isoprostanes and malondialdehyde are both byproducts of the oxidation of polyunsaturated fatty acids and are used as common biomarkers for lipid peroxidation [14]. Studies on postmortem brain samples have shown elevated levels of F2-isoprostanes in the frontal and temporal lobes and in the cerebrospinal fluid (CSF) of Alzheimer's subjects when compared with age-matched cognitively normal controls [15,16]. Additionally, some studies have also found increased serum 8-iso-prostaglandin F2α (a stereoisomer of F2-isoprostanes) and malondialdehyde levels in Alzheimer's patients when compared with normal controls [16,17]. Similar biomarkers of oxidation have been found in patients with depression as well. A recent meta-analysis found increased serum and urine levels of F2-isoprostanes and malondialdehyde in major depressive disorder patients when compared with normal controls [14].

Nucleic acid damage

Accumulation of oxidative nucleic acid damage in the brain is a hallmark of both depression and AD [18,19]. Free radicals target nuclear and mitochondrial DNA, triggering increased base modification, double-stranded breaks, and single-stranded breaks in neuronal cells, resulting in cell death [18]. Furthermore, hydrogen peroxide (H2O2) induces oxidative damage by increasing microsatellite instability in DNA [20]. Telomerase and 8-oxoguanine (8-oxoG) are promising biomarkers representative of DNA damage in neurological disorders [21].

Telomeres are repetitive DNA sequences located at the end of chromosomes that protect against DNA damage and maintain genomic stability [22]. However, in AD and depression, it is believed that oxidative stress results in the shortening of telomeres by inhibiting telomerase, telomerase reverse transcriptase, and telomerase RNA component activity, which are enzymes responsible for maintaining telomere length [22,23]. Studies on AD mouse models showed that sustained physiological expression of telomerase reverse transcriptase in adult neurons resulted in decreased amyloid aggregates and improved cognition [24]. Similarly, researchers investigated telomerase activity in the hippocampus by comparing a rat model for depression with control rats and determined that telomere length, telomerase activity, and telomerase reverse transcriptase activity were markedly decreased in the depression model [25].

8-oxoguanine, while capable of pairing with cytosine, primarily exerts its mutagenic effects through mispairing with adenine, leading to G:C→T:A transversions that initiate DNA repair mechanisms, including OGG1-mediated base excision repair, which is particularly critical in neuronal DNA maintenance [26]. Via glycosylating mechanisms, excess 8-oxoG and its derivatives are excreted in urine, making it an efficient measurable biomarker to assess oxidative stress [26]. Apart from urine, these biomarkers can also be found in blood serum, tissue, and CSF. Several studies have found elevated levels of 8-oxoG and its derivatives within urine, serum, and leukocytes of patients with depression when compared with controls [27-30]. Additionally, studies conducted on AD mouse models and the serum of Alzheimer’s patients showed significantly elevated levels of 8-oxoG when compared with healthy controls [31,32]. Some researchers have theorized that decreased base excision repair mechanisms contribute to the elevations seen in 8-oxoG and believe that oxoguanine glycosylase-1 protein could be a potential biomarker for nucleic acid damage from oxidative stress [32].

Oxidative and nitrosative stress

Proteins are essential macromolecules in the body needed to maintain the normal structure and function of cells and tissues. Oxidative modification of proteins can alter their structure and affect the normal physiological function of cells, eventually leading to cell death and tissue damage [33]. Elevated levels of reactive nitrogen species, such as peroxynitrite, lead to protein nitration that could change the primary, secondary, tertiary, or quaternary structure of a protein, thereby affecting its function [33]. Tyrosine is a non-essential amino acid that can undergo post-translational nitration to form 3-nitrotyrosine (3-NT), a biomarker for oxidative and nitrosative stress. Proteins involved in forming the neuronal cytoskeleton contain tyrosine residues that may be converted to 3-NT, and elevated 3-NT levels have been observed in experimental models of neurodegeneration [34]. Whether tyrosine nitration directly depletes tyrosine availability for dopamine synthesis or causally contributes to neuronal dysfunction in human depression or AD remains to be established. Tyrosine is also the precursor to dopamine; hence, nitrotyrosine can affect dopaminergic neurons and cause a decrease in dopamine concentrations in the brain, a pathological feature of depression [35,36]. Studies have shown significantly increased urine and serum 3-NT levels in depressed patients when compared with normal controls [36]. Similarly, studies conducted on Alzheimer’s patients have also shown significantly increased 3-NT levels in plasma, CSF, and postmortem brain samples when compared with normal controls [33,34,37]. Increased levels of 3-NT were also found in pre-tangles (abnormal cytoplasmic tau prior to fibril formation) of early Alzheimer's brains, suggesting that 3-NT could be a potential biomarker for early Alzheimer's detection [33,38].

Metal ions

In the realm of AD research, some metal ions serve as significant markers and subjects of research interest due to their participation in pathogenesis. Copper is one of these metal ions of interest, most notably when there is an elevated concentration of non-ceruloplasmin-bound copper in patients with AD, raising scientific interest regarding copper’s role in the pathogenesis and progression of AD [39]. Ceruloplasmin is known to be an α2 glycoprotein that harbors antioxidative properties and is the primary form of copper in the bloodstream [40,41]. In addition, reduced levels of ceruloplasmin have also been reported in patients with AD [42]. These findings indicate that the absence of ceruloplasmin and higher concentrations of free copper are markers of AD, suggesting that oxidative stress is a possible mechanism in the pathogenesis of AD. Copper remains a metal ion of interest in its participation in exacerbating depression-like behavior, particularly in individuals who carry the ApoE4 genetic variant. When mice that carry the ApoE4 gene are exposed to doses of copper, they are more likely to exhibit depressive behaviors in the forced swimming test [43]. These behaviors include increased immobility times, which are analogous to learned helplessness and emotional distress seen in human depression [44]. At the molecular level, copper levels are known to significantly impact catecholamine levels such as dopamine and norepinephrine. Dopamine, a mood-regulating neurotransmitter, is known to be deficient in Menke’s disease and accumulated in Wilson's disease, contributing to depressive symptoms [45]. In summary, copper research highlights the investigation of neurotransmitter disturbances in depression in comparison with oxidative stress in Alzheimer’s research.

Vascular dysfunction

Both AD and late-life depressive syndromes exhibit significant vascular involvement, impacting brain metabolism, β-amyloid clearance, and potentially preceding characteristic pathophysiological and cognitive symptoms [46,47]. However, the relationship between depression and AD is complex and encompasses distinct clinical entities: late-life depression (including vascular depression as a subtype), prodromal depressive symptoms emerging during preclinical AD, and behavioral and psychological symptoms of dementia (BPSD) that occur once dementia is established [48]. These entities differ in their mechanistic underpinnings and temporal relationship to neurodegenerative pathology, yet share common vascular substrates.

Vascular dysfunction, including alterations in vessel dynamics, angiogenesis, vascular cell function, coverage, blood-brain barrier integrity, and immune cell migration, is implicated across these phenotypes and may be linked to amyloid toxicity, oxidative stress, and apolipoprotein E (APOE) genotype [46]. These vascular deficits may contribute to parenchymal amyloid deposition, neurotoxicity, glial activation, metabolic dysfunction, and a feedback loop that worsens neuronal and vascular pathology throughout disease progression [46]. In late-life depression, particularly the vascular depression subtype, cerebral small vessel disease with white matter hyperintensities and subcortical microvascular lesions disrupts frontal-subcortical-limbic networks involved in mood regulation [47]. The endothelial layer of cerebral arterioles, a critical barrier between blood circulation and the brain, plays a significant role in both conditions [47]. Endothelial dysfunction, frequently driven by vascular inflammation, is a likely factor explaining the co-occurrence of depression and cardiovascular disorders, as well as associations with hypercholesterolemia, dyslipidemias, diabetes, obesity, hypertension, and aging [47].

Distinguishing primary late-life depression from prodromal depressive symptoms in preclinical AD and from BPSD in established dementia has important treatment implications. Prodromal depressive symptoms may emerge when a threshold of neurodegeneration is reached, reflecting underlying amyloid and tau pathology rather than representing an independent psychiatric disorder [48]. In contrast, BPSD, which includes depression, apathy, agitation, and psychosis, occurs in over 90% of individuals with AD during the disease course and reflects the interaction of cognitive impairment, neurodegeneration, and environmental factors. The coexistence of cerebral small vessel disease and AD pathology (amyloid/tau) further complicates this clinical picture, as vascular lesions may both predispose to depression and accelerate cognitive decline in those with underlying neurodegenerative disease [46,48].

Cell adhesion molecules, including intercellular adhesion molecule-1 (ICAM-1), are elevated during vascular injury or dysfunction, such as the deposition of amyloid β (Aβ) characteristic of AD [47]. Recent research comparing gross brain specimens in individuals with depression and AD shows that, in the depression group, increases in interleukin (IL)-6 and IL-10 and decreases in IL-1β were observed compared with controls, whereas in the AD group, especially early-stage, only increased ICAM-1 expression was found compared with controls [48]. This indicates that endothelial activation may be an early feature of AD and a potential means for early detection.

Mitochondrial dysfunction

Mitochondria assume pivotal roles in cellular processes such as apoptosis, ferroptosis, and inflammasome activation. Their notable involvement in neurologic disorders, particularly AD and depression, underscores their significance. Within the context of AD, mitochondrial dysfunction is marked by excessive production of ROS, disturbances in mitochondrial Ca2+ levels, and adenosine triphosphate (ATP) depletion [49]. Additionally, it disrupts essential aspects such as mitochondrial dynamics, transport, and mitophagy (mitochondrial autophagy). The sequences of mitochondrial dysregulation highlight the intricate and multifaceted impact of this dysfunction, emphasizing the critical need to map these pathways in pharmacologic research. Ongoing investigations provide substantial evidence indicating significant disruption in mitophagy during cases of depression [50]. This disruption leads to heightened levels of mitochondrial reactive oxygen species (mtROS) and the release of cell-free mitochondrial DNA. The presence of cell-free mitochondrial DNA triggers the activation of damage-associated molecular patterns within microglia, due to its similarity to bacterial DNA, setting off a state termed "sterile inflammation." This inflammatory response is characterized by the activation of the innate immune system through nuclear factor kappa B (NF-κB), NOD-like receptor family pyrin domain containing 3 (NLRP3), and TLR3 (toll-like receptor 3). In conclusion, understanding the intricate role of mitochondria in neurologic disorders, particularly AD and depression, holds immense promise for advancing targeted therapies and improving outcomes for those affected by these conditions.

Gliosis/inflammation/microglial dysfunction

Reactive gliosis is a response to injury in the central nervous system (CNS) tissue, characterized by an increased amount of microglia and astrocytes, along with glial scar formation. The first step in reactive gliosis is an increase in microglia at the site of damage [51]. Microglia are the resident macrophages of CNS tissue that secrete proinflammatory markers such as tumor necrosis factor alpha (TNF-α), IL-1β, and IL-6. When activated, overproduction of these markers may cause neurotoxicity [52]. This neuroinflammation is a key feature in both AD and major depressive disorder. AD commonly presents with depression as a symptom, along with anxiety, irritability, and other mood disorders [53]. These proinflammatory markers are thought to cause an increase in phosphorylation of abnormal tau, which appears in neurofibrillary tangles and is a hallmark of AD in the brain [54]. In addition, these pro-inflammatory cytokines serve as activators of astrocytes, which subsequently increase in size, express glial fibrillary acidic protein (GFAP), and secrete neurotrophic factors such as insulin-like growth factor (IGF), nerve growth factor (NGF), and brain-derived neurotrophic factor (BDNF) [55]. Astrocytes also secrete TNF-⍺, IL-1β, and IL-6, which, in turn, may activate microglia, further enhancing inflammation [56]. In AD, astrocytes show increased production of Aβ peptide, which is a major component of amyloid plaques [57,58].

Dysregulations of the hypothalamic-pituitary-adrenal (HPA) axis have been implicated in the impairment of learning and memory in mice [59]. In mice, Aβ1-42 oligomers have been associated with impairment of the HPA axis, leading to overactivity and greater concentrations of corticotropin-releasing hormone and corticosterone, which ultimately lead to a lowering of neuroprotective factors, resulting in cognitive impairment in AD [60]. Additionally, mice placed under stress displayed an increase in Aβ aggregates in the hippocampus and amygdala [61]. Ongoing investigation is needed to determine whether Aβ aggregates are the cause of HPA axis dysfunction or whether chronic stress leads to increased Aβ. Overactivation of the HPA axis and cortisol hypersecretion have been found in individuals with major depressive disorder [62]. In rats, repeated corticosterone injections increased depression-like behavior, suggesting that depression is related to increased corticosterone levels [63]. Further research on the relationship between the HPA axis in AD and depression is necessary to fully understand the inflammatory effects of cortisol.

Integrin proteins and signal peptides have been found to have neuroprotective effects and are targets for potential therapies for brain disease [64]. Dysfunction may lead to increased inflammation and reduced protection from certain diseases. Mendelian randomization analysis of depression and AD genes has recently found two potential proteins - integrin beta-5 (ITGB5) and signal peptide peptidase-like protein-1 - that, when mutated, may lead to immune dysregulation in the brain [65]. An important component in creating functional integrin receptors includes ITGB5, which is critical for cell migration, proliferation, differentiation, and immune response regulation. Dysregulation of ITGB5 may contribute to the pathogenesis of AD and depression and may be used as a potential therapeutic target as well [65]. Signal peptide peptidase-like protein-1 has been found to be implicated in intracellular signal transduction and protein degradation processes, reducing the accumulation of toxic proteins and ultimately providing neuroprotection [66]. When signal peptide peptidase-like protein-1 is dysfunctional, it may lead to the accumulation of toxic proteins; however, further investigation is necessary to determine the exact mechanism of action [65].

Antidepressants have been found to have anti-inflammatory effects, reducing inflammatory markers such as TNF-⍺ or IL-6, which further enhances their efficacy for mood disorders and overall brain inflammation [67]. Given the anti-inflammatory effects of antidepressants, individuals on these medications may be less likely to develop AD. Certain antidepressants, particularly tricyclic antidepressants like imipramine, have demonstrated TNF-α inhibitory properties that may be relevant to AD pathology [68]. In preclinical studies, imipramine prevented cognitive decline and reduced Aβ accumulation in mouse models of AD, effects that were mediated, in part, through inhibition of TNF-α [68]. Specifically, mice treated with imipramine (10 mg/kg) showed significant improvements in both long-term and short-term memory function, along with decreased TNF-α levels in the frontal cortex and reduced Aβ peptide accumulation in both the frontal cortex and hippocampus [68].

Efficacy of antidepressants in treating AD

Antidepressants are a frequently studied treatment option for AD. Due to the urgent need for symptom relief for AD patients, antidepressants have been explored as potential direct and indirect therapeutic remedies. One meta-analysis found seven studies that directly compared antidepressants with placebos to relieve depressive symptoms in AD. The analysis concluded no significant difference (odds ratio (OR) 1.95, 95% confidence interval (CI) 0.97-3.92) between controls and sertraline, mirtazapine, imipramine, fluoxetine, and clomipramine [69]. A more recent study by Zhang et al. found that use of escitalopram (0.813 SMD, 95% CI 0.207 to 1.419, p = 0.009), paroxetine (1.244 SMD, 95% CI 0.939 to 1.548, p < 0.000), and sertraline (0.818 SMD, 95% CI 0.274 to 1.362, p < 0.000) significantly alleviated depressive symptoms in AD patients [70].

An interesting avenue of exploration lies in repurposing antidepressants, specifically selective serotonin uptake inhibitors and serotonin antagonists and reuptake inhibitors, to alleviate the primary features of AD. Trazodone, a serotonin antagonist and reuptake inhibitor, has been shown to delay cognitive impairment in individuals diagnosed with AD. Using the Mini-Mental State Examination (MMSE), the rate of cognitive decline decreased by a factor of 2.6, to 0.27 points per year in trazodone users (95% CI 0.07-0.48) versus 0.70 (95% CI 0.50-0.90) for non-users (p = 0.023) [71]. Furthermore, a five-year retrospective study on healthy, cognitively normal older individuals showed a significant negative correlation between cumulative selective serotonin reuptake inhibitor use and Aβ levels [72]. In addition, acute administration of the selective serotonin reuptake inhibitor citalopram has been shown to significantly decrease CSF Aβ formation in both transgenic mice and healthy humans. In mice that were chronically administered citalopram, a 78% reduction in the appearance of Aβ plaque formation was noted. In healthy humans, acute administration of citalopram led to a 37% decrease in Aβ plaque formation, resulting in a 38% decrease in total Aβ CSF concentration [73]. Further clinical trials could elucidate the relationship between serotonin modulation and Aβ plaque formation in individuals with AD.

Limitations

This narrative review has several important limitations that warrant consideration. First, unlike systematic reviews, our methodology did not employ a comprehensive, pre-specified search strategy or explicit inclusion/exclusion criteria, which may have introduced selection bias in the literature reviewed. The integration of diverse evidence types, including meta-analyses, randomized controlled trials, observational studies, and mouse models, presents inherent challenges, as each study design requires different critical appraisal methods and contributes different levels of evidence to our understanding of the AD-depression relationship.

Second, significant translational gaps exist between animal models and human pathology. The mouse models discussed predominantly focus on Aβ and tau accumulation but fail to capture the full complexity of human AD, including early-stage pathologies, genetic diversity, and critical socioemotional factors that influence both depression and neurodegeneration in aging humans. Studies demonstrate that depression-related phenotypes in transgenic mice do not consistently correlate with neurobiological changes in ways that mirror human disease progression, limiting the generalizability of mechanistic insights derived from these models [74]. Third, the bidirectional and temporally complex relationship between depression and AD complicates causal inference [75,76]. Depression may function as a risk factor, prodrome, or symptom of dementia, with these relationships potentially varying across the lifespan, making it challenging to establish definitive mechanistic pathways, even when synthesizing evidence from multiple methodological approaches [75,76]. Finally, shared pathophysiological mechanisms, including neuroinflammation, HPA axis dysregulation, and neurotransmitter alterations, make it difficult to determine whether observed associations reflect common underlying biology or represent true causal relationships between these two conditions [76]. These limitations underscore the need for future systematic reviews with rigorous methodology and translational studies that better model the complexity of human aging and neuropsychiatric disease.

## Conclusions

Our paper explores the complex relationship between AD and depression by examining a multitude of biomarkers and describing how the interplay of oxidative stress, DNA damage, lipid damage, metal ions, vascular dysfunction, mitochondrial dysfunction, and microglial dysfunction is a component that links these two diseases. A key finding from this investigation is that oxidative stress, manifested through the imbalance of various antioxidants and ROS, plays an essential role in the progression of both AD and depression. Additionally, mitochondrial dysfunction contributes to the increased production of ROS, as well as cellular dysfunction seen in both diseases, which is demonstrated by disturbances in ATP and mitochondrial calcium homeostasis and loss of organelle processes, such as mitophagy. Furthermore, inappropriate cell-free mitochondrial DNA release worsens the accumulation of oxidative stress through innate immunity mechanisms involving damage-associated molecular patterns and expression of inflammatory transcription factors (e.g., NF-κB). We have also demonstrated links of metal ions, particularly copper and iron, to the progression of both AD and depression, which offer promising avenues for further investigation and potential therapeutic intervention. Additionally, vascular dysfunction appears to disrupt cellular metabolism, eventually leading to cellular injury and loss of normal function, and exacerbating the cognitive decline and mood disturbances seen in AD and depression. Studies have also found benefits from certain antidepressants, particularly selective serotonin reuptake inhibitors; however, further research is needed to determine the efficacy of antidepressants in treating concurrent AD and depression. The lack of effectiveness in current therapies for AD with depression prompts us to consider potential pharmacological interventions that target common pathways and mechanisms. Moreover, the future use of diagnostic tools that focus on the identified biomarkers may provide new insight into the management and treatment of these debilitating diseases.

In closing, we have demonstrated the overwhelming overlap between AD and depression. These biomarkers seem to put individuals at risk for both AD and depression; however, it resembles a "chicken and egg" scenario, where the exact causal sequence remains unknown. Our paper has brought to light a shared landscape of biomarkers and pathways that may connect these conditions, offering new insight for effective treatments and preventive measures. In the future, continued research will hopefully unravel the mechanisms behind the interplay between the two diseases, leading to improved outcomes for those affected by AD and depression.
